# Spatial and temporal distribution of optimal maize sowing dates in Nigeria

**DOI:** 10.1371/journal.pone.0300427

**Published:** 2024-05-02

**Authors:** Siyabusa Mkuhlani, Eduardo Garcia Bendito, Abdullahi I. Tofa, Kamaluddin Tijjani Aliyu, Bello Muhammad Shehu, Christine Kreye, Abel Chemura

**Affiliations:** 1 International Institute of Tropical Agriculture, Nairobi, Kenya; 2 International Institute of Tropical Agriculture, Kano, Nigeria; 3 International Maize and Wheat Improvement Center (CIMMYT), Lusaka, Zambia; 4 Department of Soil Science, Bayero University Kano, Rimin Gata, Nigeria; 5 International Institute for Tropical Agriculture, Ibadan, Nigeria; 6 Department of Natural Resources, Faculty of Geo-Information Science and Earth Observation, University of Twente, Enschede, Netherlands; Feroze Gandhi Degree College, INDIA

## Abstract

Climate change and inter-annual variability cause variation in rainfall commencement and cessation which has consequences for the maize growing season length and thus impact yields. This study therefore sought to determine the spatially explicit optimum maize sowing dates to enable site specific recommendations in Nigeria. Gridded weather and soil data, crop management and cultivar were used to simulate maize yield from 1981–2019 at a scale of 0.5°. A total of 37 potential sowing dates between 1 March and 7 November at an interval of 7 days for each year were evaluated. The optimum sowing date was the date which maximizes yield at harvest, keeping all other management factors constant. The results show that optimum sowing dates significantly vary across the country with northern Nigeria having notably delayed sowing dates compared to southern Nigeria which has earlier planting dates. The long-term optimal sowing dates significantly (*p*<0.05), shifted between the 1980s (1981–1990), and current (2011–2019), for most of the country. The most optimum planting dates of southern Nigeria shifted to later sowing dates while most optimum sowing dates of central and northern Nigeria shifted to earlier sowing dates. There was more variation in optimum sowing dates in the wetter than the drier agro-ecologies. Changes in climate explain changes in sowing dates in wetter agro-ecologies compared to drier agro-ecologies. The study concludes that the optimum sowing dates derived from this study and the corresponding methodology used to generate them can be used to improve cropping calendars in maize farming in Nigeria.

## Introduction

Maize (*Zea mays* L.) is one of the staple food and feed crops in Africa, providing over 60% of the calories for over 1.2 billion people. About 40.7 million hectares are under maize production in Africa and this translates to almost 100 million metric tonnes, which accounts for close to 40% of total cereal production on the continent [1]. Similarly, in Nigeria, maize is an important dietary crop, with production of at least 10 million metric tonnes and cultivated on at least 6 million hectares of arable land [2]. It is consumed at least once on a daily basis by an average Nigerian citizen [3]. Maize farming is practiced across the country in small, medium, or large-scale farms and employs many people in the production and post-production value chains [4, 5].

There are multiple factors affecting maize crop production, such as low soil fertility, limited access to inputs, literacy gaps, poor infrastructure and access to markets and climate change and variability [6]. In recent years, climate change and variability have become major variables influencing maize crop yields and production in Africa [7] particularly because the majority of the maize production is rainfed [6]. Climate variability is increasingly becoming a threat to crop production in Sub-Saharan Africa (SSA) for the past three decades [8]. In Nigeria, variability in rainfall onset, cessation and length of growing season variability have also increased due to climate change [9]. The impacts are more notable in semi-arid to arid agro-ecologies, although they are widespread across all regions. Climate change has led to coefficient of variabilities which are as high as 98.1% in rainfall onset in Kenya, during the period 1979–2009. Growing season rainfall variability of up to 33.4–68.1%, has also been recorded. Consequently, maize yield variability has ranged from 42–78.3%, with maize yields varying from 44.5 kg/ha to 4830 kg/ha over the period 1979–2009 [7]. In Ghana climate variability explains maize yield variations of 4.2, 22.5, 39.2 and 23.1% through changes in rainfall, soil moisture, minimum and maximum temperature for the period, 1981–2015 [10].

Smallholder farmers who are characterised by limited resource endowment, have challenges in using some practices such as irrigation to counteract the impacts of climate change [11]. In SSA, a significant number of studies on climate change impacts and variability management have been conducted [12, 13]. These studies have shown that several climate smart agricultural practices that can be utilised to enhance climate change adaptation [12, 13]. Despite the proven notable increase in yields, the uptake of such practices has been low. This is attributed to the capital required to change farming practices to implement such climate smart agricultural practices [14]. As a result, low-cost climate smart agriculture practises such as optimizing sowing dates, among others, need to be evaluated.

Missing planting opportunities can lead to significant maize yield losses of up to 5% per week’s delay [15]. This is potentially caused by in season dry spells and pest and diseases outbreaks coinciding with early crop reproductive stages. Early sowing, especially in Northen Nigeria, leads to poor germination and ultimately low yields due to seed weather damage, soil pests and loss of seed viability. On the other hand, delayed planting potentially results in crop failure, as the crop runs out of soil moisture due to cessation of rains before passing critical phenological and developmental stages [16, 17].

Use of optimal planting dates can potentially result in yield gains in maize of up to 43% in southern Africa [18] and in Nigeria [19, 20]. Evaluation of planting dates has been undertaken using on station field trials. Such studies are however complex and expensive to undertake. Several approaches that have been used for planting dates assessment are remote sensing [21], indigenous knowledge [22], crop models [18, 20], and rainfall seasonality analysis [9].

Recent studies on assessing planting dates have used crop modelling approaches [20, 23–25], due to their ability to simulate crop growth and crop yield levels by means of different input variables, particularly soil and weather information, under various management options. However, most of the modelling studies to determine planting dates have been carried out at specific locations and were then generalized for a larger target area; which might not closely resemble the modelled site. The increased temporal and regional climate variability may render such an approach potentially ineffective. The optimal dates for sowing may therefore vary over smaller geographic areas and subsequent years. This study therefore sought to determine the optimum sowing time at national scale for Nigeria using a spatially gridded modelling approach for four decades. Specifically, the study sought to determine (1) the optimum sowing date for maize in different agro-ecological zones of Nigeria, (2) evaluate temporal changes in sowing date at a spatial scale of 0.5 ° for maize in Nigeria and (3) determine how the changes in sowing dates varied across agroecological zones.

## Materials and methods

### Study locations

Nigeria comprises of 7 agro-ecologies from humid forest to sahel savannah. The agro-ecological zones have considerable differences in rainfall, temperature and growing season length which has a notable impact on the vegetation and predominant crops (Table 1).

**Table 1:** Bio-physical characteristics of different agro-ecologies of Nigeria [26, 27].

Agro-ecology zone	Total annual rainfall (mm)	Annual mean temperature (°C)	Growing season length (days)	Vegetation	Main crop	Growing period (days)
Humid forest	2000–3000	25–27	270–360	Forest	Cocoa Oil palm	300–360
Derived Guinea savannah	1500–2000	26–28	211–270	Forest	Oil palm Yam Maize	200–250
Southern Guinea savannah	1200–1500	26–29	181–210	Savannah	Yam Maize Sorghum Soybean Sesame	150–200
Northern guinea	900–1200	27–29	151–180	Savannah	Maize Sorghum Soybean Cotton	150–200
Sudan savannah	500–900	25–30	91–150	Savannah	Millet Sorghum Groundnut	90–150
Sahel savanna	250–500	21–32	<90	Grassland	Millet Sorghum	>90
Mid-high altitude	1100–1500	20–23	160–200	Savannah	Maize Potato Vegetable	200–300

### Field experiments

The study used the Decision Support System for Agrotechnology Transfer (DSSAT) CERES-Maize crop model which was calibrated and validated, for maize based on-field data from Zaria and Iburu, in Kaduna State, Nigeria [20]. The modelling work was based on field work undertaken by researchers from the International Institute of Tropical Agriculture (IITA), which has been officially accredited by the Federal Republic of Nigeria, to undertake agricultural research in Nigeria since 1967 [28]. The DSSAT CERES maize crop simulation model is a dynamic process-based crop model that can simulate crop growth, development [29]. The model was calibrated based on the five-year period (2014–2019), while the evaluation was based on the 2015 and 2016 seasons. The experiments used for point-based model calibration were laid out in a randomised complete block design (RCBD), with maize varieties of different growing duration used as treatments which were replicated 3 times. The modelling research used IWDC2Syn-F2-W, a single medium maturing variety, as this provided a representative variety, as opposed to short or long seasoned varieties. The experimental plot had 4 rows of 5 m in length. The inter and intra row spacing was 0.75 and 0.25 m apart. Basal dressing (N: P: K, 15:15:15), was applied at a rate of 60 N, 60 kg P_2_O_5_ and 60 kg K_2_O kg/ha one week after planting. A top dressing of 60 kg N/ha was applied 45 days after planting through Urea (46% N). The experimental plots were kept weed free using post-emergent herbicides. Manual hoe weeding was carried out at 4 and 8 weeks after planting [20].

Measurements undertaken from the experimental plots were maize phenology, grain, and biomass yields. Days to 50% tasseling was taken as parameter for flowering, while days to maturity was measured when 95% of the population reached physiological maturity. The harvested grains were dried, and yield calculations adjusted to 12% moisture after measuring the grains moisture content. Results for the phenology (flowering and maturity), grain, and biomass yields were previously reported by Tofa et al. [20]. Rainfall, temperature, and solar radiation were measured at a daily time step using the automated WatchDog^®^ 2000 series weather station device located at the experimental sites. Soil profile pits were dug to determine the morphological, physical and chemical properties. Soil texture, organic carbon, total nitrogen, pH and available phosphorus were measured using standard laboratory methods.

The RMSE for phenology, grain and biomass yields was less than 30% which was within the acceptable range. A RMSE value of less than 10% was considered excellent, 10–20% ‘good’, ‘20–30%’ fair and above 30% ‘poor’ [30]. The index of agreement values were all above 0.6 meaning there was considerable agreement [30]. The index of agreement represents the ratio of the mean error and the potential error, where a value of 1 represents a perfect match and 0 means no agreement at all. A model forecasting efficiency value closer to 1 is more skilful. The model forecast efficiency for all the parameters was above 0.75 which was an indication of the model’s ability to mimic what was observed on the ground [20].

The correlation coefficient (r) and coefficient of determination (R^2^), root mean square error (RMSE, Eq 1, percent bias (pBias (%), Eq 2), and index of agreement (d, Eq 3), were therefore used to assess the model performance.


$$\text{RMSE}=\sqrt{\frac{1}{n}}\sum {\left(y_{i}-\hat{y}\right)}^{2}$$ (1)

$$\text{pBias}=\left(\frac{\sum \left(y_{i}-{\hat{y}}_{i}\right)*100}{\sum y_{i}}\right)$$ (2)

$$d=1\;-\left[\frac{\sum_{i=1}^{n}{\left({\hat{y}}_{i}-y_{i}\right)}^{2}}{\sum_{i=1}^{n}{\left(\left|{\hat{y}}_{i}-\bar{y}\right|+\left|y_{i}-\bar{y}\right|\right)}^{2}}\right].$$ (3)

For all the cases n is the number of data points, yi and ŷi denote the reference and simulated yield, and ȳ is the mean of the observed yield (Moriasi et al., 2007).

### Spatial model simulations

The point based calibrated and evaluated DSSAT CERES-maize model [20], was utilised to undertake spatial maize yield simulations for sowing dates for the window 1 March to 7 November, with sowing taking place every 7 days, with other management practices being the same. This was undertaken for each year, from 1981–2019. The sowing period was chosen since it is the main maize growing season for maize in Nigeria. The simulations were undertaken at a spatial scale of approximately 50*50 km across the whole country. The DSSAT crop model [29], was enabled to run in a spatialised form through automated linking of the input and DSSAT source code using R programming language [31]. Gridded weather from W5E5 [32] and soil data from ISRIC [33], were used as input data for model simulations. The W5E5 data was selected for this study because the data has a complete set of climate parameters (rainfall, maximum temperature, minimum temperature, radiation), which are the basic parameters needed for the crop model to run. The ISRIC data was selected because it provides a reliable soil dataset for use in spatial modelling frameworks and has been widely used in similar studies. The weather and soil data were formatted from a general data frame into a DSSAT input model ready format using the R programming language.

The yield outputs from the model simulations were normalised to a scale of 0 to 1. This was undertaken because there was extreme variability in the data. This allows data to be brought into a more comparable range, to have relevant comparisons. Data normalisation was undertaken using log function in R [31].

### Model evaluation

To determine the reliability of the spatially simulated maize yields, we evaluated the model performance with reference maize yield data at the same grid scale of 0.5*0.5°. We compared the simulated maize yield with the maize yield from the Spatial Production Allocation Model (SPAM, 2017), yield data [34] to evaluate whether the simulated yield data closely matched the resampled SPAM yield data to 0.5 ° grid. The evaluation compared grid data from our simulations and those from SPAM. The SPAM data has been widely used in gridded modelling studies because it provides farming system specific yields from a collection of sub-national statistical data integrating ancillary information including crop prices, population density and crop-specific biophysical suitability to distribute sub-national statistics within the crop [35–38], land extent using cross entropy.

The point based parameterized and calibrated model was applied to independently simulate the maize yield for the period 1981 to 2019 with the W5E5 observational climate data. To evaluate the model, we checked for correspondence of the DSSAT maize yields for 2017, with the selected year of 2017 maize yields from SPAM. We used the correlation coefficient (r) and coefficient of determination (R^2^), root mean square error (RMSE, Eq 1, percent bias (pBias (%), Eq 2) and index of agreement (d, Eq 3) to assess the model performance.

### Data analysis

The analysis was undertaken using R based statistical functions [31]. The optimum planting date is the date where the yield is the highest. The optimum planting dates in each pixel for each of the years during the period, (1981–2019), were evaluated for significant changes in the optimum sowing date trends, using the Man-Kendall test [39, 40]. Further analysis was undertaken to evaluate inter-decadal changes in optimum sowing date, to improve understanding of the changes in sowing dates as well as changes within and across agro-ecological regions. We also compared our optimal sowing dates with the publicly available Center for Sustainability and the Global Environment (SAGE) sowing dates (https://sage.nelson.wisc.edu/data-and-models/datasets/crop-calendar-dataset/arcinfo-ascii-5-min/) to determine how much the current sowing dates deviate from the optimal sowing dates from our model [41].

## Results

### Model evaluation

**Fig 1:**
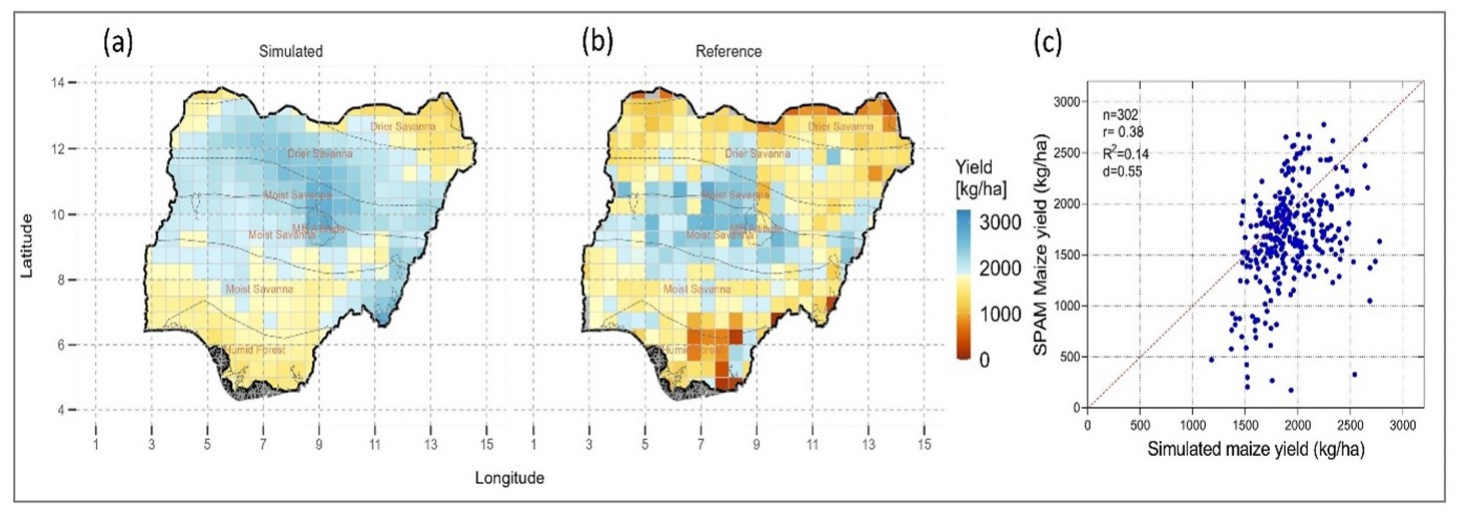
(a) DSSAT crop model simulated maize yield for 2017, (b) SPAM yield for 2017, and (c) comparison across the agro-ecological zones in Nigeria. © Authors. Source-Shapefile: https://data.humdata.org/dataset/nga-administrative-boundaries.

The simulated maize yield, (corresponding to the optimum sowing date) was compared with the SPAM reference yield for Nigeria for the year 2017 which had corresponding yield data. The model was able to capture the general gradient in maize yield in the country with lower yields in the south, north-east and north-western parts of the country and higher yields in the central parts forming the moist savanna agroecological region (Fig 1a and 1b). However, the simulated yields were higher than the reference yield for most areas and also the extend of maize potential extended slightly that of the observed maize yield from the reference data. The highest model fit was for the Arid/Sahel (d = 0.59) agroecological region followed by the Semi-arid/Sudan savanna (d = 0.53) and Southern Guinea savanna (d = 0.51), (S1 File). Overall, the model performance was satisfactory with an index of agreement of 0.55 (Fig 1c).

### Optimum planting date

**Fig 2:**
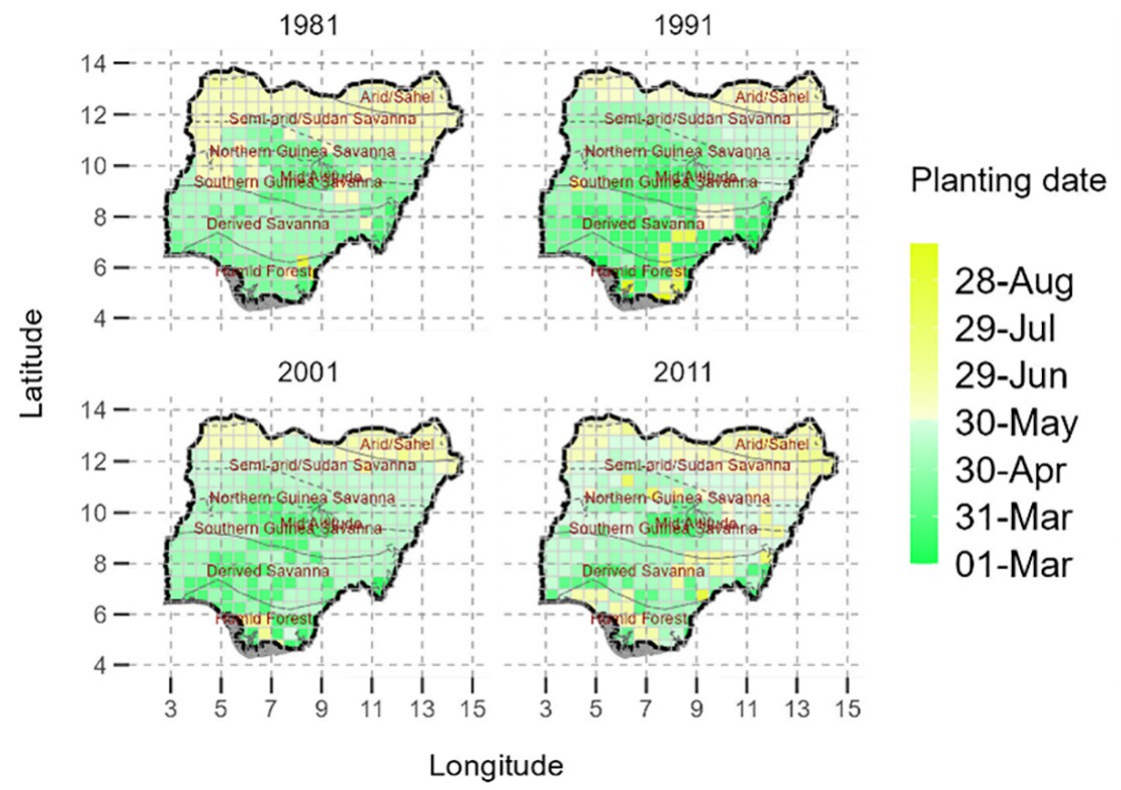
Distribution of optimal sowing dates across Nigeria for the period 1981, 1991, 2001 and 2011. © Authors. Source-Shapefile: https://data.humdata.org/dataset/nga-administrative-boundaries.

Results show that optimum sowing dates in Nigeria ranged from March 1 to November 6 for the period 1981–2019, with a latitudinal delay in optimal sowing dates from the south to the north (Fig 2). Optimum sowing dates are earlier in southern Nigeria and delayed in northern Nigeria. Specifically, the optimum sowing dates in the northern regions are delayed to as much as around 29 July. In the agro-ecological regions found in southern Nigeria, the earliest optimum sowing dates were as early as 10-April. The mid altitude and some areas in the southern region have the earliest optimum sowing dates of around 1 March (S1 File).

With regards to agro-ecologies, the drier savanna in northern Nigeria is generally characterised by later optimum sowing dates than the humid forest, moist savanna and mid altitude agro-ecologies (S1 File). In the arid/sahel region the mean optimal sowing date was 16 June. This was notably different to the sowing date realised in the mid-altitude agro-ecologies which had an average optimal sowing date of 15-April. The optimal sowing dates in the savannas were all within a period of 1 month (3-May to 5-June), with derived savannah having an optimal date of 3-May and semi-arid savannah having a sowing date of 5-June (Table 2).

**Table 2:** Mean optimal sowing dates of maize across the different agro-ecologies in Nigeria for the period, 1981–2019.

Agro-ecological zone	Mean planting date
Arid/Sahel	June 16^a^
Semi-arid/Sudan Savanna	June 5^b^
Northern Guinea Savanna	May 19^c^
Southern Guinea Savanna	May 15^d^
Derived Savanna	May 3^e^
Humid Forest	April 30^f^
Mild Altitude	April 15^g^

The pattern however does not hold for a few seasons for example in 1981 where for the entire North of Nigeria (25% of Nigeria), the optimum sowing dates were relatively late in the season between 1 to 30 June. There were a few regions where the optimum sowing dates were between 1 March to 10 April. There were only two grid boxes with 6-Sept as the optimum sowing date. Most of the country has an optimum sowing date of between 10-April to 30 May except for the years 1991, 2013, where almost 90% of the country had optimum dates of between 1-April and 30 June, though the sowing dates are still delayed for northern Nigeria relative to the southern Nigeria. About 6 grid boxes (2%), in the south and south-east of Nigeria have notably delayed sowing dates of around 7-Sepetember (S1 File).

### Long term optimum planting date trends

**Fig 3:**
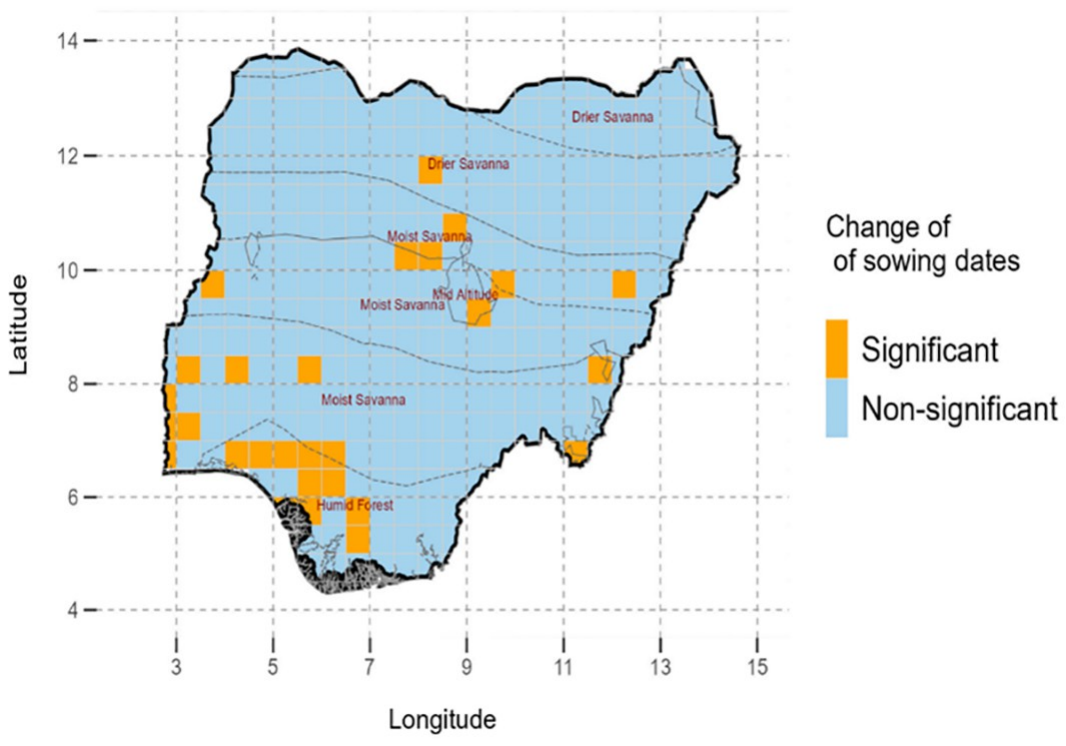
Average long-term changes in sowing dates and their level of significance from 1981–2019 in Nigeria. © Authors. Source-Shapefile: https://data.humdata.org/dataset/nga-administrative-boundaries.

The study assessed the long-term trends in the optimal sowing dates for each grid box for 39 years (Fig 3). The study showed for most (91%), of Nigeria there were no significant changes in optimal sowing dates from 1981 to 2019. Changes were only noted in about 8% of the country, which is generally in South-west and north central Nigeria regions. Specifically, the changes were noted in the humid forest and moist savanna and only one grid box constituting less than 1% of the drier savanna (Fig 3).

**Fig 4:**
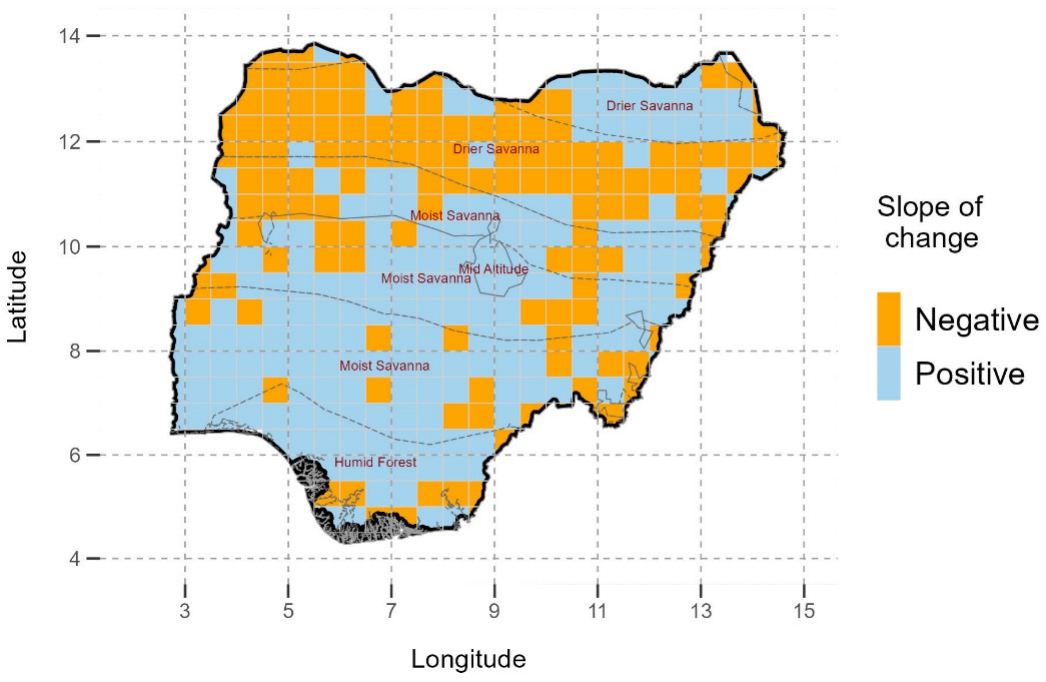
General direction of average changes in sowing dates from 1981–2019 in Nigeria. © Authors. Source-Shapefile: https://data.humdata.org/dataset/nga-administrative-boundaries.

With regards to the general direction of change in optimum sowing date, about 60% of the country showed increased delay in sowing dates over the last 39 years. Notably 40% of the country showed a negative slope in change in the sowing dates. Specifically, Northern Nigeria which is dominated by the drier savanna showed predominantly a negative slope, meaning sowing dates were changing from relatively late to earlier. On the contrary in humid forests, moist savanna and mid altitude the sowing dates changed from relatively earlier to later as realised by the positive slope, with potential implications on decreasing the length of the growing season (Fig 4).

### Agro-ecology specific changes in sowing dates

**Fig 5:**
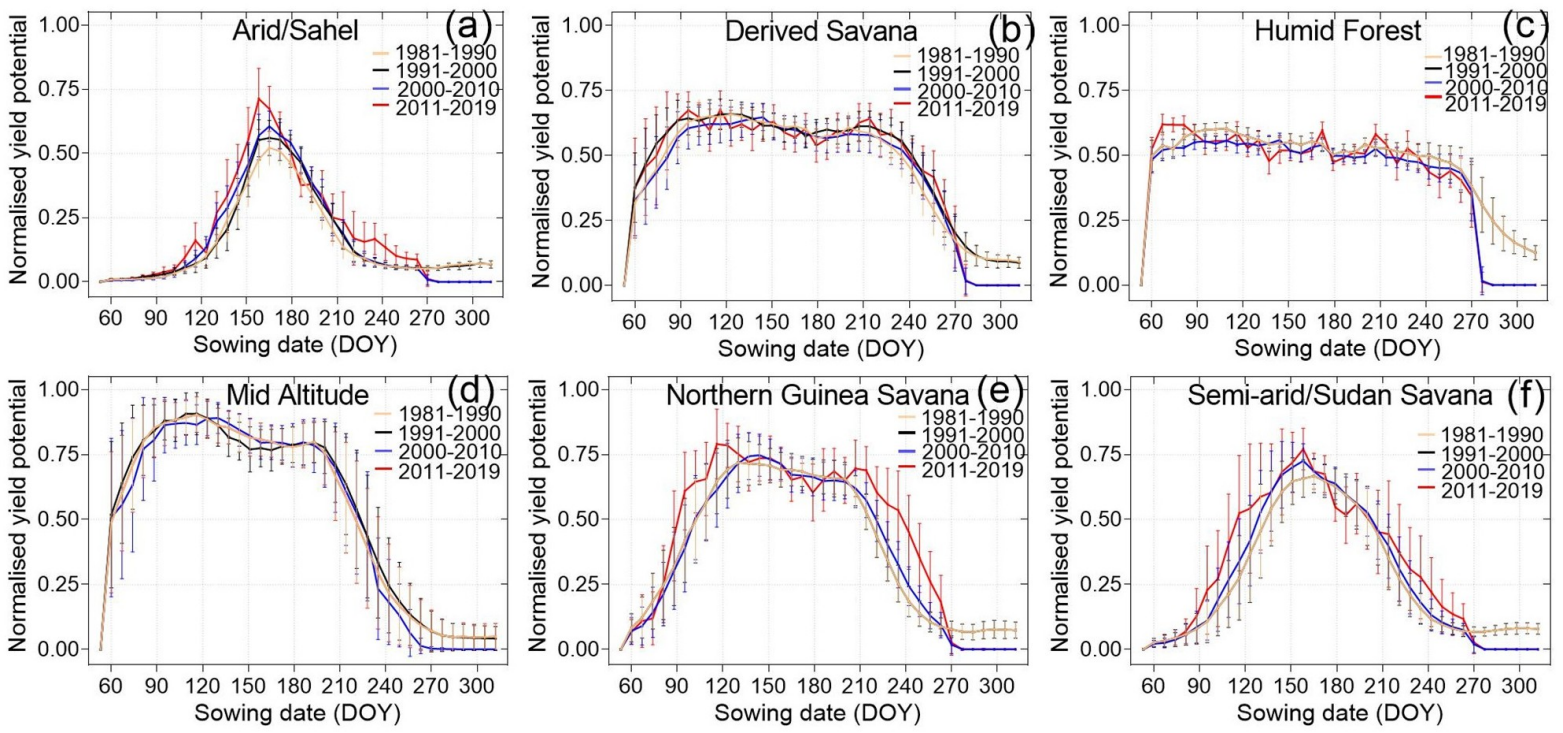
Yield penalties from sowing dates by agroecological region and their decadal shifts in sowing dates in Nigeria obtained from the simulation. © Authors.

Over the past four decades there have been changes in the optimum sowing dates in the different agro-ecologies and between decades. Generally, compared to the 1980s, optimum sowing dates in the 1990s showed reduction in the optimum dates, meaning the optimum sowing dates were earlier in the year compared to the 1980s (Fig 5).

**Fig 6:**
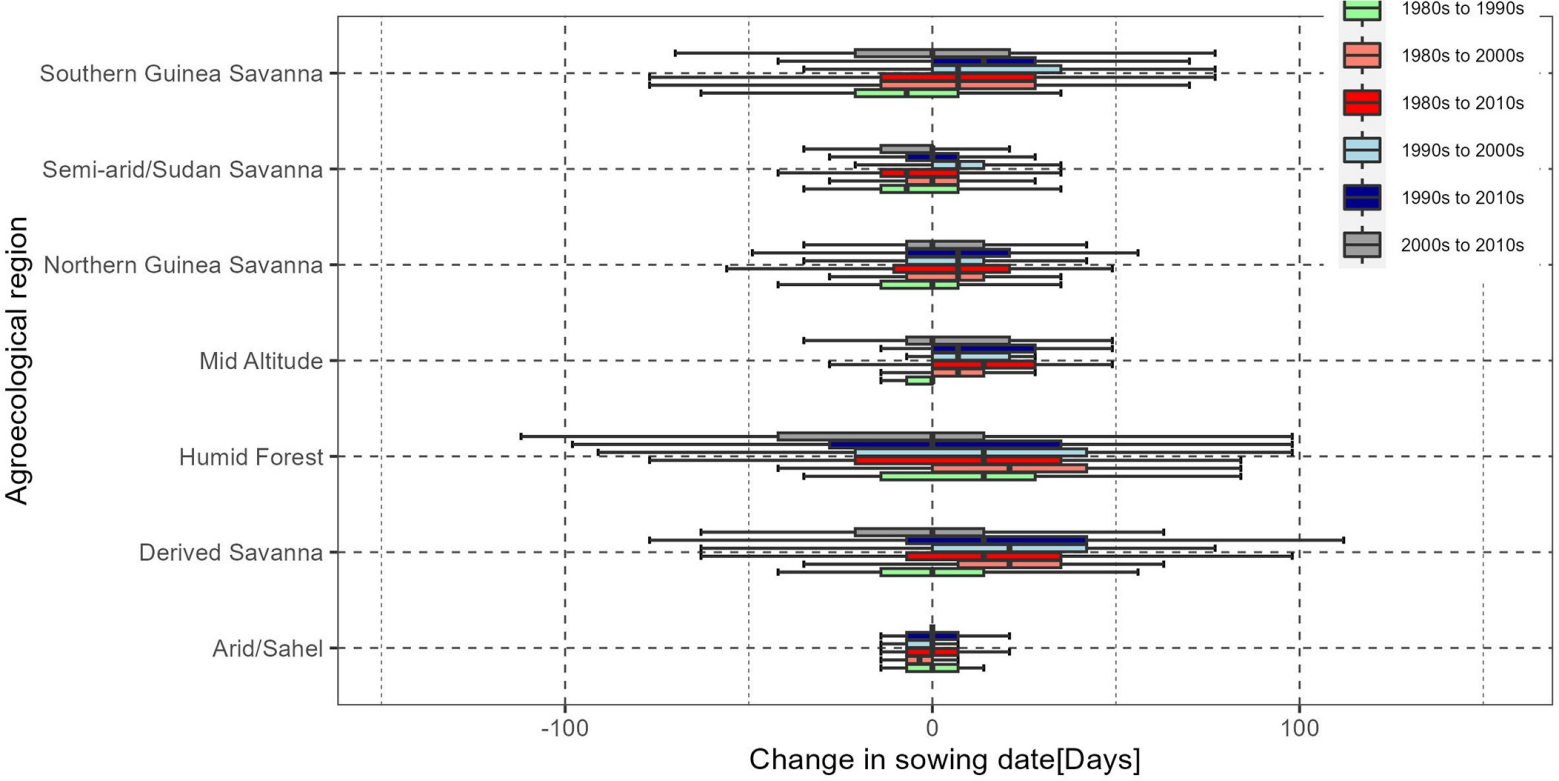
Agro-ecology specific long-term changes in sowing dates for the period 1981 to 2019. © Authors.

Specifically, there was notable variation in changes in interdecadal changes in optimum sowing dates in all agro-ecologies except in the arid/Sahel agro-ecologies. The changes in the optimum sowing dates in arid/Sahel agro-ecology were the least at an average of +/-7 days. In Semi-arid/Sudan savanna the changes were also relatively low at +/– 13 days (Fig 6). There was notable delay in optimum sowing dates of up to 13 days during the period 1990s and 2010s, compared to the 1980s. Compared to the 1990s, the sowing dates in the 2010s increased by up to 13 days (Fig 6), (S1 File). In the mid altitudes the average changes were mostly earlier, extending up to 30 days for most interdecadal comparison.

The results show that there were severe yield penalties in missing optimal sowing dates in the Arid/Sahel agroecological region and the semi-arid/Sudan savanna region as yield potential declines sharply from the specific optimal date (Fig 5a and 5f), (S1 File). We observe that the trends in the optimal sowing dates are that they are becoming earlier in the mid altitude, the Semi-arid/Sudan savanna and the arid/Sahel agro-ecologies in the more recent decades. Interestingly, in the northern Guinea savanna agroecological region, the first sowing window is becoming earlier while the second sowing window is becoming later, as indicated by a stretch in the windows at the two ends (Fig 5e).

Notable changes in sowing dates were realised in the derived savanna, humid forest and southern Guinea savanna agro-ecological regions. The increases in sowing date which denotes delay in sowing dates were notable for humid forests and derived savanna. Specifically, there were increases and decreases of almost 40 days in the humid forest. In the derived savannah, the changes ranged from -63 to 100 days (Fig 6). In the Southern Guinea savanna, the changes were +/- 75 days. For the 3 agro-ecological zones, when compared to the 1980s, 2010 led to delayed sowing dates by 50–100 days. This was generally similar for the 2010s compared to 1990s and 2000s, compared to the 1980s period (Fig 6).

### Comparison with the reference sowing date data

**Fig 7:**
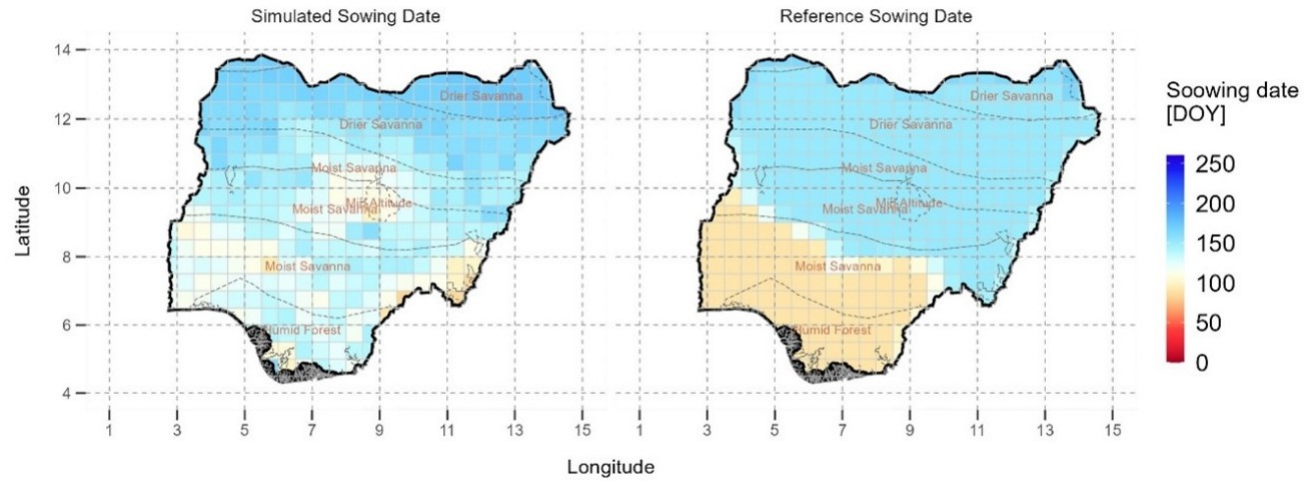
Comparison of the optimal simulated and reference Center for Sustainability and the Global Environment (SAGE), 2010, maize sowing dates in Nigeria. © Authors. Source-Shapefile: https://data.humdata.org/dataset/nga-administrative-boundaries.

We compared the optimal sowing dates with reference data from SAGE that show the observed maize sowing dates for the period around year 2000. We find that there is a big mismatch between the reference sowing dates and the identified optimal sowing dates in Nigeria, except for the northern areas (Fig 7). The reference sowing dates data completely misses the earlier potential sowing that is possible in the mid-altitude agroecological zone and also recommends delayed sowing in the south-eastern parts of the country compared to the simulated sowing dates. A large area in the SE and SW of the country is also simulated to have specific earlier sowing dates than those in the reference data, with a difference of between 21 and 49 days.

## Discussion

### General goals

The goal of this study was to predict the spatial and temporal variability in sowing dates for maize, an important crop in Nigeria in the face of climate change and variability. This was done to in one hand assess if and to what extend sowing dates are changing and to provide a precursor to an approach that in future could be used to develop a dashboard of site-specific optimal sowing dates for maize farmers in the country. This is because deciding optimal sowing dates is recognized as an important agronomic measure to reduce the impacts of climate change. Changing sowing dates is a low-cost method to manage climate risk and increase yields as opposed to other methods [42–44]. It is therefore hoped that, when coupled with provision of information in a gridded form and sufficient confidence on model performance, such information provides a strong basis for integration of sowing dates into the basket of agronomic options, updating maize cropping calendars in the country and building farmer resilience, productivity and profitability.

### Spatial variability of sowing dates

The study realized that generally sowing dates vary across Nigeria, from the north to the south. Northern Nigeria has notably later sowing dates in the season compared to Southern Nigeria where the sowing dates are earlier in the season. This trend is generally attributed to the higher rainfall and lower variability experienced in the Southern parts of the country, which also increases the chances of having an early optimum sowing date. It has been established that the length of the growing season is critical for yield outcomes as it determines how much time the crop can grow and complete its required physiological stages, within the growing season [45–47]. Early sowing potentially leads to a relatively longer time for the plant to accumulate dry matter, which leads to higher yields as long as the conditions that are required for each crop stage are sufficient [48]. Earlier sowing also elongates the period under which the crop can utilize available water for crop growth and development, leading to high yields. As noted by [44], earlier sowing dates in March and April lead to the highest maize yields which decreases with delayed planting until June as they produce early and better flowering and taller plants with higher ear placement that contribute to higher yields. In this study we therefore observe a notable association between early sowing dates in the growing season and relatively high maize yields in Nigeria.

On the contrary, delayed sowing shortens the period the crop is exposed to ideal conditions such as rainfall, solar radiation and temperatures and this therefore increases climate risks before the crop is stronger [49]. However, there are also some risks associated with early sowing such as early or mid-season dry spells or other bio-physical challenges whose impact on the crops could be severe as these might affect the crop when it is not well established [50, 51]. This could be attributed to dry spells that are increasingly becoming a threat for maize yields in Nigeria [52]. In trying to manage such risk, results indicate the benefit of delayed sowing dates, which is mostly common in northern Nigeria due to relatively low and highly variable rainfall. This therefore increases the chances of delayed sowing dates so as to enable sowing to be undertaken under conditions of no immediate threat of dry spells [45, 53]. Therefore, our study underscores the need to identify the optimal sowing dates as they have a direct relationship on the crop yields and therefore it is critical for farmers in Nigeria to be able to explore the optimal sowing time to achieve optimal crop yields including in-season precipitation.

The changes in sowing dates in the southern part of Nigeria can potentially be attributed to an average decrease in rainfall of about 5mm/year. This is associated with the general increase of the Sea Surface Temperatures (SSTs) over the Gulf of Guinea [54, 55]. On the contrary, changes in northern Nigeria, can be attributed to, the increase in SSTs over the Mediterranean and adjacent oceans, which weakens dry northerly winds penetrating Northern Nigeria. This gradually increases rainfall by about 2–4 mm/year [55]. On the other hand, the small changes in the optimal sowing dates realized in the arid/Sahel agro-ecological region can potentially be attributed to the already very arid conditions with limited further changes hence the sowing dates will not vary notably.

### Temporal variation in sowing dates

On assessment of the optimal sowing dates over a specific point over the entire period, the study indicates that in over 75% of the country there were no significant changes in the optimum sowing date over the four decades. This finding is significant in two distinct ways. First, studies on sowing dates in Nigeria [44, 56, 57] and elsewhere [46, 48, 49, 58], mainly rely on few seasons of site-constrained experimental trials which cannot show long term trends in sowing dates, particularly in the face of a changing climate. Secondly, and perhaps most importantly, our study provides, for the first time, an impact assessment of climate change on maize sowing dates using a long time -series at a large scale for the whole of Nigeria. The current discourse on shifting sowing dates in climate impacts discussions in Nigeria is based on anecdotal evidence, farmers’ recollections of general practices in decades past and in some cases innuendos. It is therefore quite significant that we provide a sound scientific basis of trends in sowing dates and their spatial distribution in Nigeria for impact reporting and agronomic decision support.

Our finding that significant changes are only in a quarter of the country is contrary to the common assumption that due to climate change there will be notable changes in commencement of rainy season and mid-season dry spells which also affects the sowing dates leading to delayed sowing dates. However, the areas where we find significant shifts in sowing dates are part of the major maize producing areas in the humid savanna and moist savannah, which are wetter regions as compared to the arid and semi-arid regions. This is also in contrary to the common narrative that notable climate change impacts leading to changes in sowing dates would be experienced in the already drier regions [59, 60]. Other scholars suggest that the absence of changes in sowing dates in the semi-arid regions is partially attributed to the fact that (i) these are already severely water-limited environments and (ii) are projected to have relative increases in rainfall in drier agro-ecologies [8]. The long term non-significant changes in sowing dates for 75% of the country provide an opening for the findings of the study to be used for farmer decision support. Farmers can therefore regularly sow on these dates to attain optimum yields. There is, however, need to disaggregate the sowing dates by rainfall season type e.g., short, medium and long. This can therefore disaggregate optimal sowing dates by season, hence improving the quality of decision support. Delayed sowing dates may facilitate crop establishment during the narrower onset of rains, demonstrating the benefit of delayed planting on crop establishment and growth [19, 53] This pattern is also more notable in the relatively low rainfall years, where the optimal sowing dates are delayed for the whole of Nigeria [59, 60]. This therefore signifies the need to delay sowing in seasons where the predictions show low rainfall. On the contrary, under high rainfall forecasts, the sowing dates are relatively early.

### Model performance

The model was initially calibrated and validated based on experimental data for a single location. This was extrapolated to the whole of Nigeria and this included the variety and management, conditions. Ideally the model should be calibrated and validated based on the different locations being simulated for. Such a study should be undertaken with optimal variety and management conditions for each environment as the choice of variety can have a significant interactive effect on attained yield [19, 44, 49]. Future simulations should therefore consider this to capture the genotypic and environmental interactions in sowing dates. The confidence in this study is improved though the use of medium maturity varieties, and the management practices which were obtained from previous research undertaken in the country [20]. Despite such moderate confidence, there is need for the recommendations to be validated, to ascertain their effectiveness. Validation can be undertaken through on farm surveys or field experiments. This also calls for a need for increased investment in the field and agronomy experiments to collect data, that can sufficiently feed into such research.

## Conclusions

The goal of this study was to assess if and where sowing dates have significantly changed over time in Nigeria given the notable changes in climate over four decades (1981–2019). From the findings of the study, it is confirmed that there is indeed maize growth and productivity response to variation in planting date, with degree of response varying between varying temporal and spatial scales across Nigeria. This supports the fact that using optimal planting dates maximizes maize yield and production in Nigeria. In addition, we conclude that with the long-time series analyzed, significant changes in sowing dates have only occurred in limited areas (~25% of the country), but in parts of the main maize producing areas. The general observed pattern is that sowing dates are notably later in the season in northern compared to southern Nigeria, which has an impact on the length of the growing season and productivity. The optimum maize sowing dates resulting from this study can therefore be utilized for decision making (e.g.: updating crop-calendars), as there is notable stability in the optimum sowing dates. The study also showed that sowing dates are more stable in the semi-arid agro-ecologies compared to the wetter agro-ecologies, which might be attributed to the already precarious conditions. This indicates the criticality of accurately optimized sowing dates in Northern Nigeria to not exacerbate food security risks. Further studies on season length changes and varietal responses over time are required to further understand other dynamics in maize growth and yield. There is therefore continued need for regular assessments of the climate patterns and sowing dates for wetter areas as there were notable observations in increased variability. Despite the challenges with seasonal forecasts, farmers would however need to make these decisions with additional information on seasonal forecasts.

## Supporting information

S1 File Figures on a)-model accuracy assessment for each agroecological zone, b)-historical optimum maize sowing dates for the period, 1981–2019 in Nigeria, and c) decadal change in the sowing dates for the period, 1981–2019 in Nigeria obtained from the simulation.
(DOCX)
