# Supplementary material for: Spatial and temporal distribution of optimal maize sowing dates in Nigeria
Source: PLoS One. 2024 May 2;19(5):e0300427. doi: 10.1371/journal.pone.0300427 (PMC11065300; doi:10.1371/journal.pone.0300427)
Supplement: S1 File — Figures on a)-model accuracy assessment for each agroecological zone, b)-historical optimum maize sowing dates for the period, 1981–2019 in Nigeria, and c) decadal change in the sowing dates for the period, 1981–2019 in Nigeria obtained from the simulation. (DOCX) [file pone.0300427.s001.docx]

**Supplementary information**

**
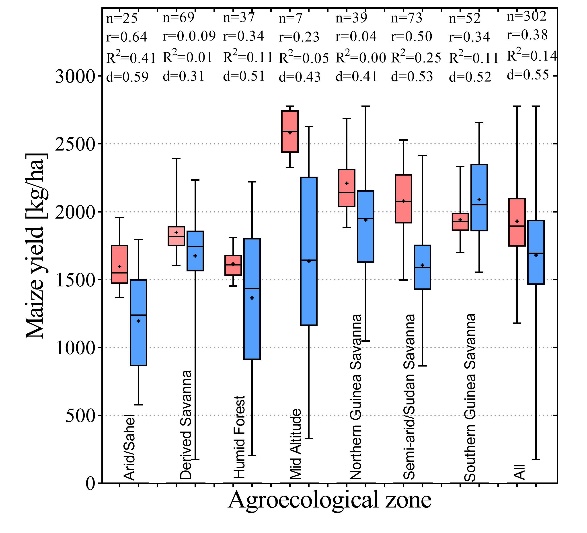
**

a) Model accuracy assessment for each agroecological zone. © Authors


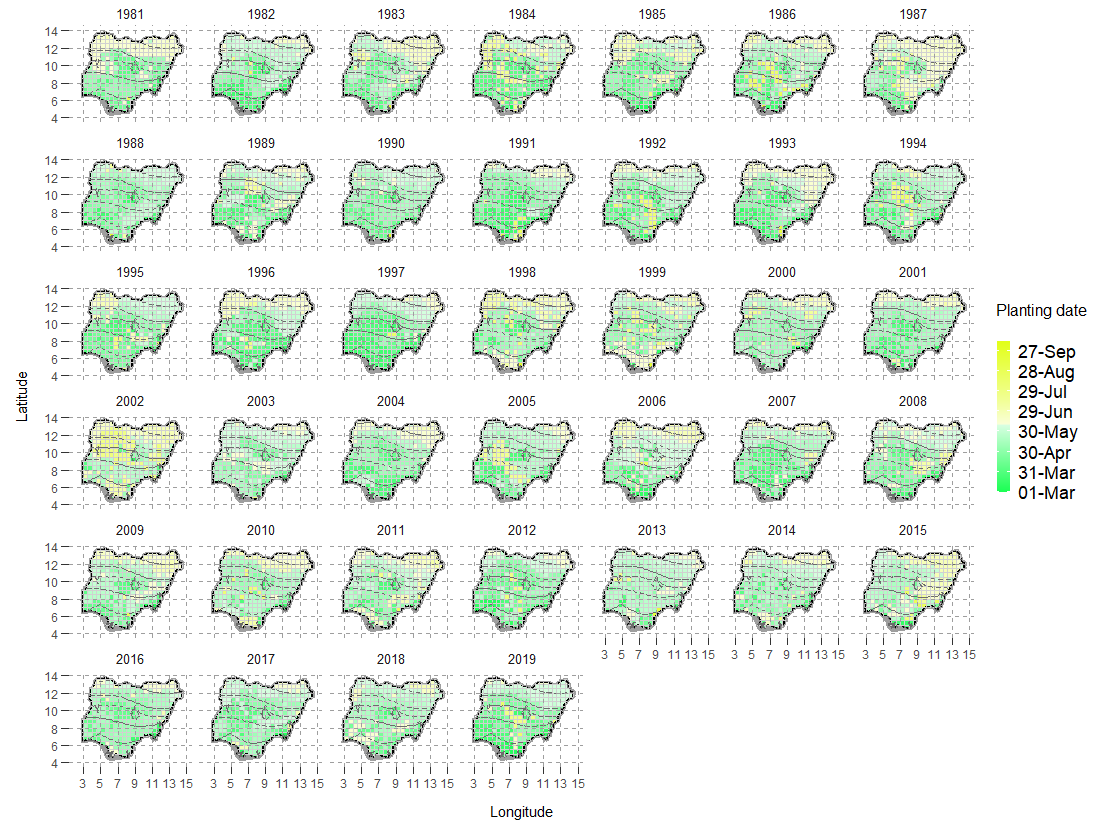


b) Historical optimum maize sowing dates for the period, 1981-2019 in Nigeria. © Authors. Source-Shapefile: https://data.humdata.org/dataset/nga-administrative-boundaries© Authors


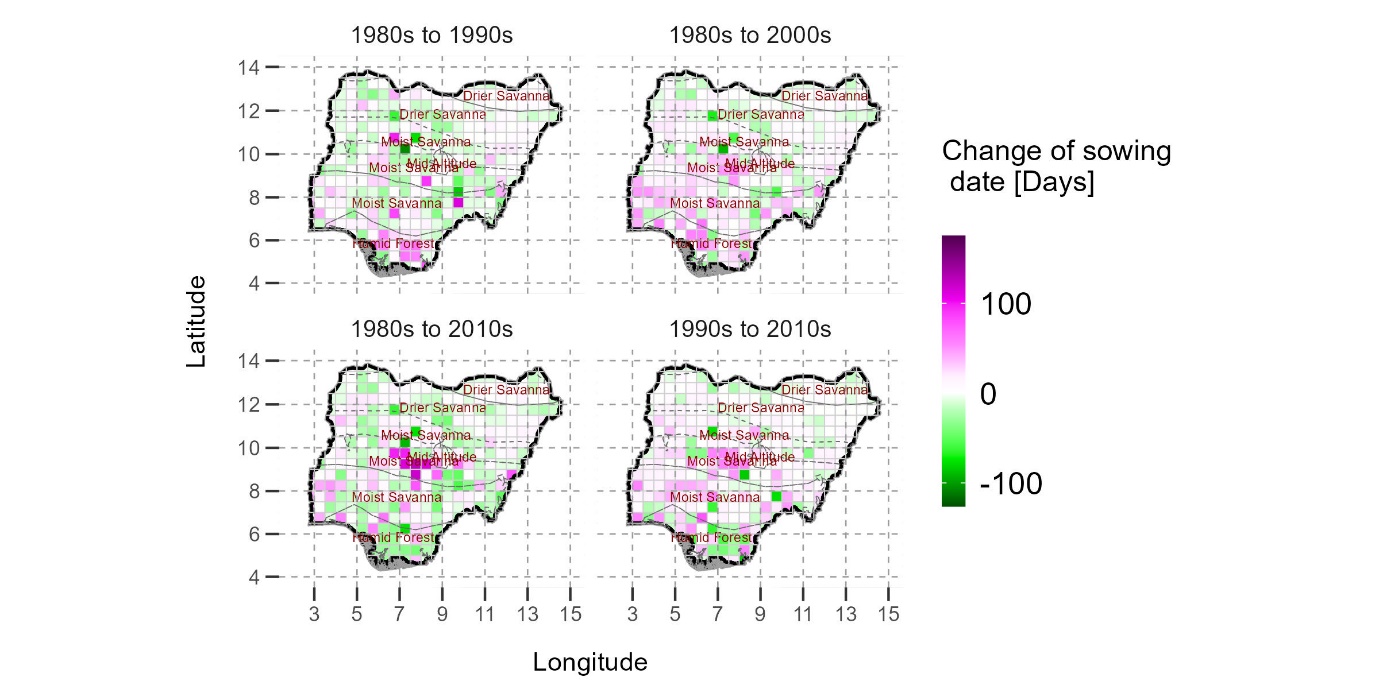


c) Decadal change in the sowing dates for the period, 1981-2019 in Nigeria obtained from the simulation. © Authors. Source-Shapefile: https://data.humdata.org/dataset/nga-administrative-boundaries© Authors
